# The bioavailability and airway clearance of the steroid component of budesonide/formoterol and salmeterol/fluticasone after inhaled administration in patients with COPD and healthy subjects: a randomized controlled trial

**DOI:** 10.1186/1465-9921-10-104

**Published:** 2009-10-31

**Authors:** Chris Dalby, Tomasz Polanowski, Thomas Larsson, Lars Borgström, Staffan Edsbäcker, Tim W Harrison

**Affiliations:** 1 Respiratory Medicine Unit, City Hospital Campus, Nottingham University, Nottingham, UK; 2 AstraZeneca R&D, Lund, Sweden

## Abstract

**Background:**

Airway absorption and bioavailability of inhaled corticosteroids (ICSs) may be influenced by differences in pharmacokinetic properties such as lipophilicity and patient characteristics such as lung function. This study aimed to further investigate and clarify the distribution of budesonide and fluticasone in patients with severe chronic obstructive pulmonary disease (COPD) by measuring the systemic availability and sputum concentration of budesonide and fluticasone, administered via combination inhalers with the respective long-acting β_2_-agonists, formoterol and salmeterol.

**Methods:**

This was a randomized, double-blind, double-dummy, two-way crossover, multicenter study. Following a run-in period, 28 patients with severe COPD (mean age 65 years, mean forced expiratory volume in 1 second [FEV_1_] 37.5% predicted normal) and 27 healthy subjects (mean age 31 years, FEV_1 _103.3% predicted normal) received two single-dose treatments of budesonide/formoterol (400/12 μg) and salmeterol/fluticasone (50/500 μg), separated by a 4–14-day washout period. ICS concentrations were measured over 10 hours post-inhalation in plasma in all subjects, and over 6 hours in spontaneously expectorated sputum in COPD patients. The primary end point was the area under the curve (AUC) of budesonide and fluticasone plasma concentrations in COPD patients relative to healthy subjects.

**Results:**

Mean plasma AUC values were lower in COPD patients versus healthy subjects for budesonide (3.07 μM·hr versus 6.21 μM·hr) and fluticasone (0.84 μM·hr versus 1.50 μM·hr), and the dose-adjusted AUC (geometric mean) ratios in healthy subjects and patients with severe COPD for plasma budesonide and fluticasone were similar (2.02 versus 1.80; primary end point). In COPD patients, the T_max _and the mean residence time in the systemic circulation were shorter for budesonide versus fluticasone (15.5 min versus 50.8 min and 4.41 hrs versus 12.78 hrs, respectively) and C_max _was higher (1.08 μM versus 0.09 μM). The amount of expectorated fluticasone (percentage of estimated lung-deposited dose) in sputum over 6 hours was significantly higher versus budesonide (ratio 5.21; p = 0.006). Both treatments were well tolerated.

**Conclusion:**

The relative systemic availabilities of budesonide and fluticasone between patients with severe COPD and healthy subjects were similar. In patients with COPD, a larger fraction of fluticasone was expectorated in the sputum as compared with budesonide.

**Trial registration:**

**Trial registration number **NCT00379028

## Background

Chronic obstructive pulmonary disease (COPD) is a preventable and treatable disease associated with considerable and increasing morbidity and mortality worldwide [1,2]. It is characterized by progressive airflow limitation that is not fully reversible [1]. Inhaled corticosteroids (ICSs) in combination with a long-acting β_2_-agonist (LABA) are recommended for the treatment of patients with severe COPD and a history of repeated exacerbations [1,3]. Two such combinations, budesonide/formoterol and salmeterol/fluticasone, are licensed for use in COPD and a number of randomized, double-blind clinical studies have demonstrated improvements in lung function and reduced numbers of exacerbations with their use [4-7].

Although these combinations both contain an ICS and a LABA, differences exist with regard to the pharmacokinetic and pharmacodynamic properties of both components, such as the oral bioavailability and clearance, volume of distribution and speed of airway uptake, which may impact on the clinical efficacy and safety of the treatments. The degree of lipophilicity, for example, varies widely. Budesonide is several times less lipophilic than fluticasone and, as a result, dissolves more readily in airway mucus and is more rapidly absorbed into the airway tissue and systemic circulation [8-10]. Fluticasone, being more lipophilic and thus less water soluble, is more likely to be retained in the lumen of the airways and therefore, has a greater chance of being removed from the airways by mucociliary clearance and cough [11]. These differences in lipophilicity may be particularly relevant in patients with severe COPD because marked airflow obstruction will lead to greater proximal deposition of inhaled drugs [12] and therefore mucociliary clearance. Indeed, previous studies in patients with asthma and airflow obstruction have shown that the systemic exposure of fluticasone is more affected by lung function than budesonide [13,14].

This is the first study to investigate and clarify the absorption of the two ICSs, budesonide and fluticasone delivered via ICS/LABA combination products, in patients with severe COPD and healthy subjects. The novel aspect of the study is the assessment of the proportion of ICS that is expectorated in sputum in patients with severe COPD.

## Methods

### Study subjects

Subjects were either healthy, as determined by medical history, physical examination, vital signs, electrocardiogram and clinical laboratory tests, or diagnosed with severe COPD. The inclusion criteria for patients with severe COPD were: aged ≥ 40 years, COPD symptoms for ≥ 1 year, a smoking history of ≥ 10 pack-years, pre-bronchodilatory forced expiratory volume in 1 second (FEV_1_) ≤ 55% of predicted normal, FEV_1_/vital capacity (VC) ≤ 70%, a productive cough with expectoration at least twice before noon on most days, and stable symptoms with no signs of an infection or COPD exacerbation within 1 month prior to study start. Exclusion criteria included asthma and/or rhinitis before the age of 40 years and use of β-blocking agents.

Healthy subjects aged ≥ 18 years with a pre-bronchodilatory FEV_1 _≥ 80% of predicted normal and an FEV_1_/VC > 70% were eligible for enrollment. Healthy subjects had to have never been regular smokers and were excluded if they were judged to have any significant illness or were using any prescribed medication, or over-the-counter remedies (except for oral contraceptives), herbal preparations, vitamins and mineral supplements ≤ 2 weeks prior to enrollment.

All subjects gave written informed consent to the study which was conducted in accordance with the Declaration of Helsinki and Good Clinical Practice guidelines, and approved by independent ethics committees.

### Study design

This was a double-blind, double-dummy, randomized, two-way crossover, single-dose, multicenter study (ClinicalTrials.gov number NCT00379028). Severe COPD patients were enrolled in Germany (one center), the United Kingdom (one center) and Sweden (one center); healthy subjects were enrolled at one center in Sweden. The first subject was enrolled on 4 September 2006 and the last subject completed the study on 22 July 2007.

COPD patients and healthy subjects attended the clinic at the beginning and end of run-in (visits 1–2). Informed consent was obtained at visit 1 and spirometry (FEV_1_) was performed at visit 2, from 2 to 8 days before visit 3 (start of the study drug administration). Forty-eight hours prior to visit 2, and throughout the study from then on, COPD patients using ICS or ICS/LABA combination therapies (budesonide/formoterol or salmeterol/fluticasone) were switched to equivalent doses of beclomethasone dipropionate (BDP). Use of other corticosteroids (including nasal and oral) was not permitted throughout the study. Patients were also not allowed to use long-acting anticholinergics, e.g. tiotropium 48 hours prior to visit 2 and throughout the study. Healthy subjects were instructed that no concomitant medication was permitted, except at the discretion of the study investigator.

**Figure 1:**
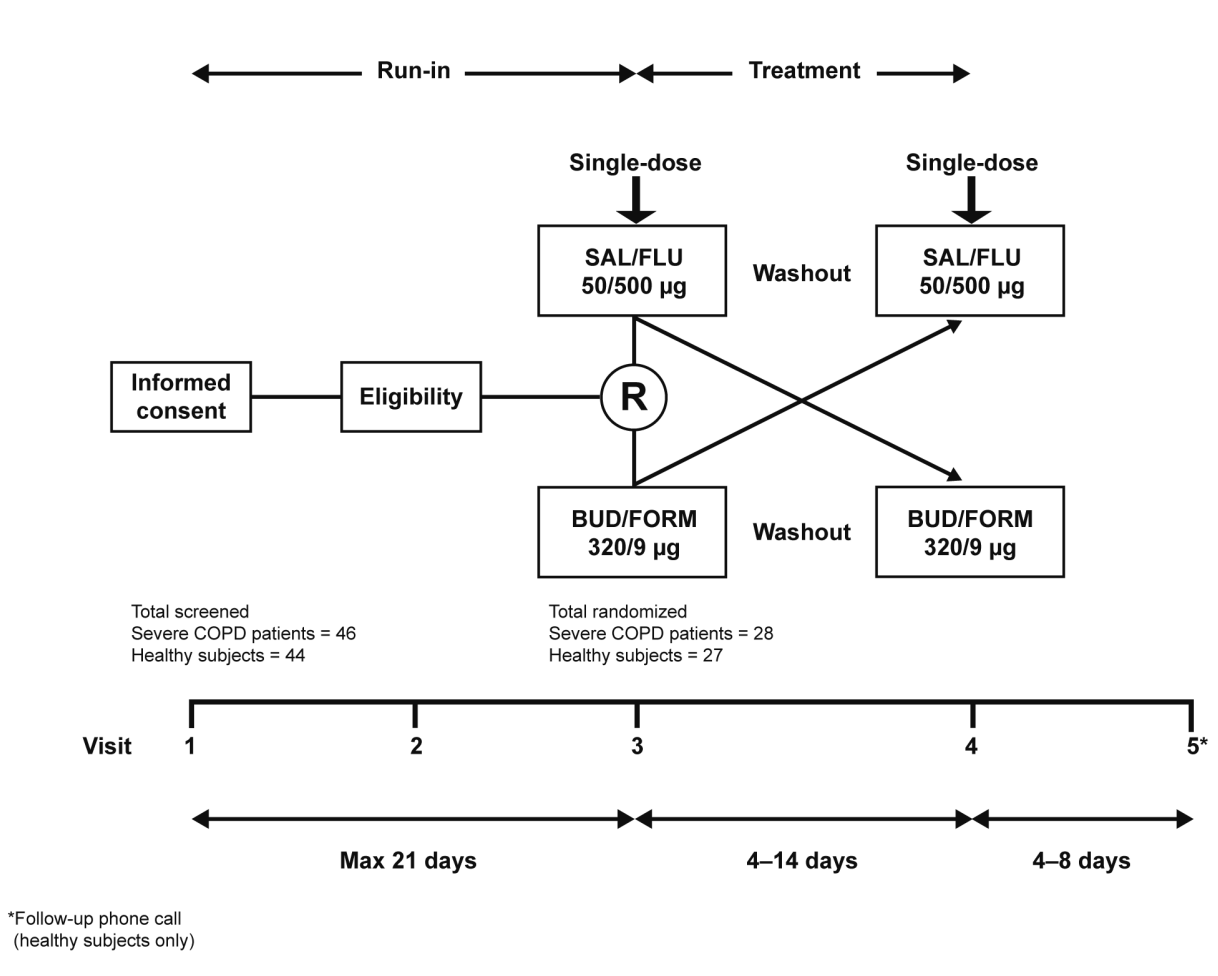
**Crossover study design**. BUD/FORM = budesonide/formoterol; SAL/FLU = salmeterol/fluticasone; R = randomization.

Following run-in, eligible participants were randomized to the treatment sequence. At each treatment visit (visits 3 and 4), study participants received, in random order, one inhalation of either budesonide/formoterol (Symbicort^® ^Turbuhaler^®^, AstraZeneca, Lund, Sweden) 400/12 μg (metered dose) plus placebo by Diskus™ (GlaxoSmithKline, Middlesex, UK) or salmeterol/fluticasone (Seretide™ Diskus, GlaxoSmithKline, Middlesex, UK) 50/500 μg plus placebo by Turbuhaler (Figure 1). COPD patients were not permitted to use BDP at either treatment visit. All participants were instructed and trained by the study investigator or nurse on the correct inhalation technique, and study drugs were administered at the same time point on both treatment visits ± 30 minutes. Each treatment visit was separated by a washout period of 4–14 days.

Randomization codes were assigned in balanced blocks from a computer-generated list at AstraZeneca Research and Development, Södertälje. At each center, participants were randomized strictly sequentially as they became eligible.

### Assessments

The primary objective was to evaluate airway tissue availabilities of budesonide and fluticasone in patients with severe COPD, using the area under the curve (AUC) of the plasma concentrations for budesonide and fluticasone in COPD patients relative to healthy subjects as a surrogate marker for airway tissue availability.

In patients with severe COPD, secondary objectives included investigating the amounts of budesonide and fluticasone spontaneously expectorated in sputum (percentage of estimated lung-deposited dose [ELDD]) and the correlation between weight of sputum expectorated, lung function and the AUCs for budesonide and fluticasone.

Blood samples for measuring the pharmacokinetic variables (AUC, maximum plasma concentration (C_max_), time for maximum plasma concentration (T_max_) and mean residence time [MRT]) of inhaled budesonide and fluticasone in plasma were obtained from all study participants via an indwelling plastic catheter in the forearm at pre-decided time points; before (at any time point between arrival at the clinic in the morning and inhalation of study drug) and at 10, 20, 40 and 60 minutes, and 2, 4, 6, 8 and 10 hours post-inhalation of the study drug at visits 3 and 4. The validated budesonide and fluticasone assays were based on a combined method of liquid chromatography-mass spectrometry (LC-MS/MS).

Since the pharmacokinetics of budesonide and fluticasone differ markedly (i.e., the uptake of fluticasone over the lung to the circulation is slower than for budesonide and the volume of distribution higher versus budesonide [14,15]), healthy subjects were used as a control.

Spontaneously expectorated sputum was collected from severe COPD patients over seven time intervals for up to 6 hours (0–10, 10–20, 20–40, 40–60, 60–120, 120–240, and 240–360 min) after study drug inhalation. Samples from each time interval were pooled, frozen immediately and stored at -20°C until further processing. After thawing, the entire expectorate was homogenized using an energetic ultrasonification treatment in combination with 0.1% dithiothreitol, as previously described [16]. Analysis of the liquidized sputum was performed using an LC-MS/MS method to measure concentrations of budesonide and fluticasone propionate. The method was validated according to the principles of the FDA Guidance for Industry Bioanalytical Method Validation [17]. The assay had a coefficient of variance at lower limit of quantification of ≤ ± 20%, in accordance with the FDA Guidelines [17] and lower and upper limits of detection of 5 nM and 10,000 nM respectively for budesonide, and 5 nM and 100 nM respectively for fluticasone.

### Statistical analysis

All hypothesis testing was done using two-sided alternative hypotheses with *P*-values < 5% considered statistically significant. Based on data from previous studies, the inter-individual (between-subject) standard deviation for the ratio of AUC between budesonide and fluticasone in healthy subjects has been estimated to be 0.29 (pooled) on the logarithmic scale [8].

Assuming a similar variation among severe COPD patients, a total of 24 patients per group was required to give 90% power to detect a 24% reduction (fluticasone expected to give a lower ratio than budesonide) in the ratio of AUC (analyzed in a multiplicative model) between COPD patients and healthy subjects.

The primary end point (AUC for budesonide and fluticasone) was assessed by a multiplicative linear mixed-effect model, with subject as a random factor and treatment, period, group (severe COPD patient or healthy subject) and treatment-group interaction as fixed factors, which was fitted to the individual dose-adjusted AUCs of fluticasone and budesonide plasma concentrations.

The relative systemic bioavailability of each ICS was estimated from this model for patients with severe COPD and healthy subjects, and expressed as the mean AUC ratio (dose-adjusted) between fluticasone and budesonide. To address the primary objective, the systemic exposure of fluticasone and budesonide was estimated from the model as the mean ratio for the dose-adjusted AUC between fluticasone and budesonide in severe COPD patients, and the mean ratio for dose-adjusted AUC between fluticasone and budesonide in healthy subjects. The associated 95% confidence intervals (CIs) were calculated.

The concentrations of budesonide and fluticasone in the expectorated sputum samples during 6 hours post-inhalation (percentage of the ELDD) were compared in severe COPD patients using a similar model, with treatment, period and patient as fixed factors. The correlation between drug-adjusted AUC and the amount of expectorated sputum for each ICS was investigated using linear regression on log AUCs and log sputum weights. The lung-delivered doses of both steroids were calculated by assuming an ELDD that was 40% of nominal dose for Turbuhaler and 15% of nominal dose for Diskus [8].

Safety outcomes were described using descriptive statistics. Safety analyses were performed on all patients who inhaled one dose or more of the study drug (full analysis set).

## Results

### Patient characteristics

**Figure 2:**
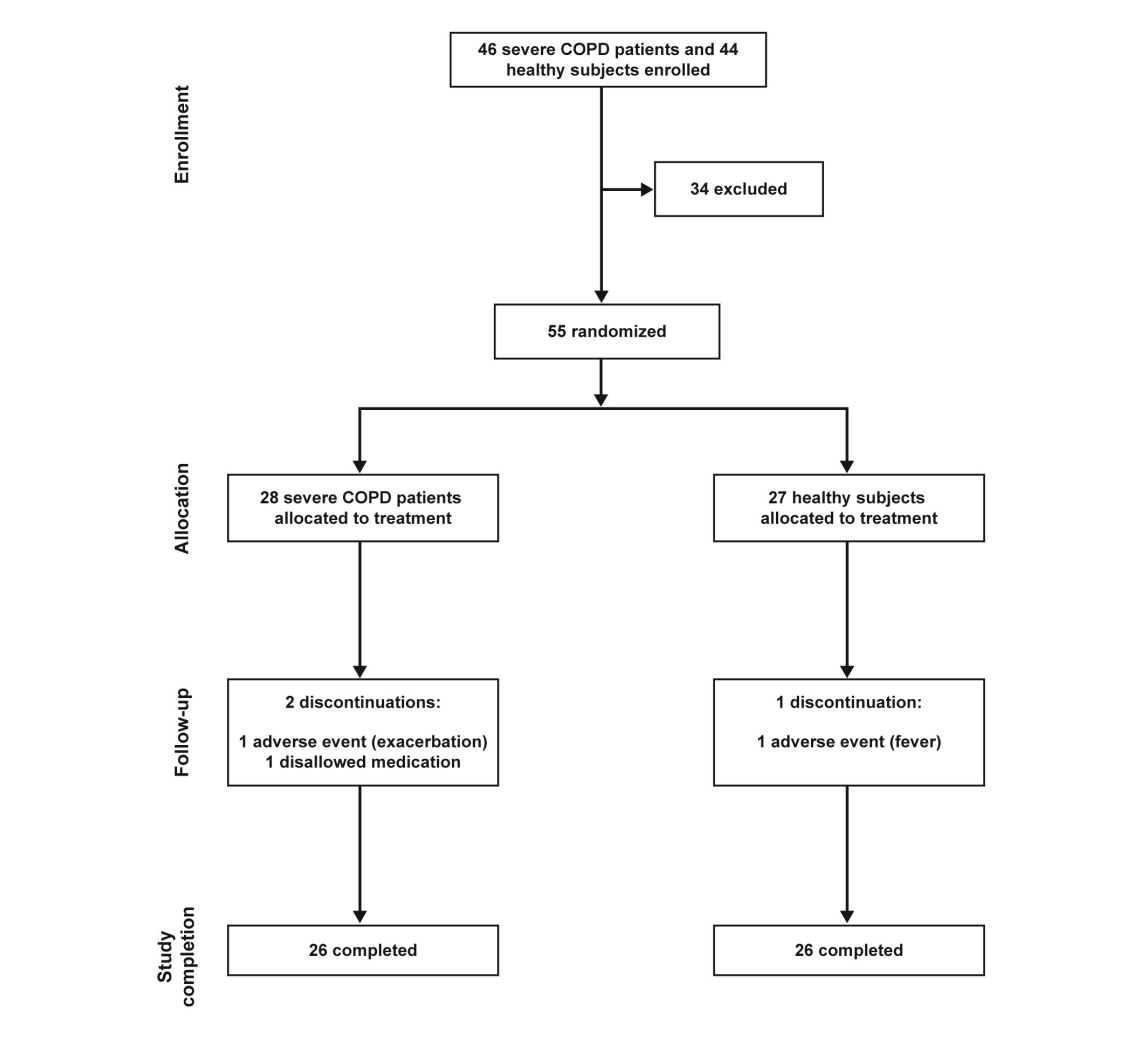
**Patient flow**.

Forty-six COPD patients and 44 healthy subjects were enrolled for the study. Twenty-eight COPD patients (mean baseline FEV_1 _37.5% predicted normal) and 27 healthy subjects (mean baseline FEV_1 _103.3% predicted normal) were randomized (Figure 2). During the study, three subjects (5%) withdrew after randomization (two COPD patients and one healthy subject).

A greater proportion of severe COPD patients were male (75%) compared with healthy subjects (41%) (Table 1). Patients with severe COPD were also older and had a higher body mass index.

**Table 1:** Demographics and baseline characteristics

	Treatment group	
	Severe COPD patients n = 28	Healthy subjects* n = 27
Male, n (%)	21 (75)	11 (41)
Age, years	65 (48-80)	31 (20-65)
BMI, kg/m^2^	26.5 (21-32)	23.1 (18-29)
FEV_1_, l	1.10 (0.5-1.9)	3.8 (2.3-5.9)
FEV_1_, % PN	37.5 (24-51)	103.3 (84-131)
VC, l	2.8 (1.2-5.2)	4.6 (3.5-6.6)
FVC, l	2.7 (1.1-4.9)	-
FEV_1_, % FVC	42.4 (27-60)	-
FEV_1_, % VC	41.6 (26-63)	83.1 (66-103)
Median time since diagnosis, years (range)	8.8 (1-37)	-
Median pack-years (range)	40 (10-64)	-
Smoking status		-
Previous, n	16	-
Habitual, n	22	-
Inhaled ICS at entry		
n	18	-
μg/day	777 (160-1600)	-

Data are mean (range) unless otherwise indicated. BMI = body mass index; FEV_1 _= forced expiratory volume in 1 second; FVC = forced vital capacity; ICS = inhaled corticosteroid; PN = predicted normal; VC = vital capacity. * Data not collected in healthy subjects on FVC, FEV_1_, time since diagnosis (not applicable [NA]), smoking (NA) and ICS (NA) at study entry.

### Systemic availability of budesonide and fluticasone

The mean plasma AUC values were lower in COPD patients versus healthy subjects for budesonide (3.07 μM·hr versus 6.21 μM·hr) and fluticasone (0.84 μM·hr versus 1.50 μM·hr) (Table 2A). The dose-adjusted AUC (geometric mean) ratios in healthy subjects and patients with severe COPD for plasma budesonide and fluticasone were similar (2.02 versus 1.80; primary end point) (Table 2B). The healthy subjects/severe COPD patient ratio of the fluticasone/budesonide ratios was estimated to be 89%, which was not significant between the drugs.

**Table 2:** Systemic availability of budesonide and fluticasone

A)				
**ICS**	**Subject group**	**n**	**AUC (μM·hr) geometric mean**	**CV**

Budesonide	Healthy subjects	24	6.21	32.7
	Severe COPD patients	24	3.07	106.4
Fluticasone	Healthy subjects	26	1.50	42.5
	Severe COPD patients	23	0.84	46.0

**B)**				

**Parameter**	**Dose-adjusted AUC geometric mean ratio**		**95% CI**	

HS/COPD for BUD	2.02		1.48, 2.76	
HS/COPD for FLU	1.80		1.32, 2.45	
FLU/BUD for HS/COPD	0.89		0.58, 1.37	

Summary of A) geometric means of area under the curve (AUC) for budesonide and fluticasone and B) geometric mean ratios for dose-adjusted AUC

BUD = budesonide; CI = confidence interval; CV = coefficient of variation; FLU = fluticasone; HS = healthy subjects.

### Pharmacokinetics of budesonide and fluticasone

**Figure 3:**
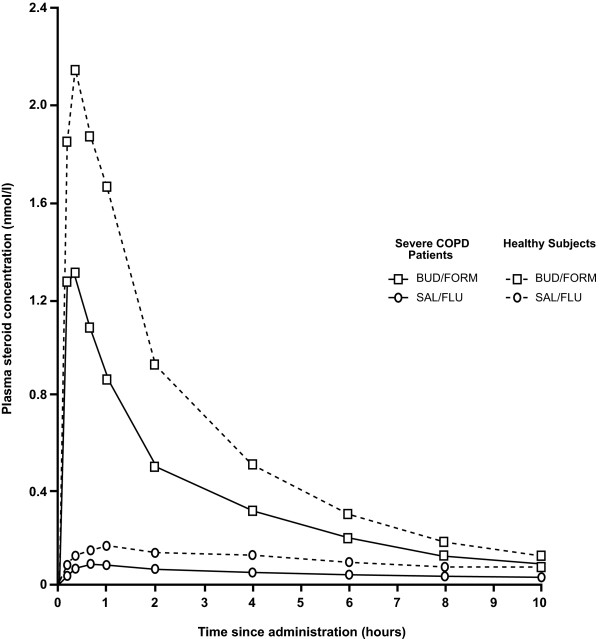
**Mean plasma concentration of budesonide and fluticasone over 10-hour sampling period in severe COPD patients and healthy subjects**. Mean (geometric) plasma concentration of budesonide and fluticasone after a single inhalation of budesonide/formoterol (BUD/FORM) (squares) and salmeterol/fluticasone (SAL/FLU) (circles), respectively, in severe COPD patients (solid lines) and healthy subjects (dashed lines).

The pharmacokinetics of budesonide and fluticasone differed from one another and between the two study populations investigated. In the patients with severe COPD, budesonide showed a fast uptake from the airways (Figure 3) with a T_max _of 15.5 min compared with 50.8 min for fluticasone, and a C_max _of 1.08 μM compared with 0.09 μM for fluticasone (Table 3). In addition, budesonide had a lower MRT in the systemic circulation compared with fluticasone (4.41 hrs versus 12.78 hrs, respectively) in severe COPD patients. In the COPD patients, the plasma concentration curve showed a more distinct peak for budesonide than for fluticasone and a similar substance difference was seen in healthy subjects (Figure 3). However, there was a tendency for both ICSs to appear in lower concentrations in severe COPD patients than in healthy subjects (Figure 3, Table 2).

**Table 3:** Summary of pharmacokinetic parameters in plasma for severe COPD patients

	Treatment	n	Mean	SD/CV
T_max _(min)	Budesonide	24	15.5*	7.2*
	Fluticasone	23	50.8*	25.4*
MRT (h)	Budesonide	24	4.41*	1.59*
	Fluticasone	23	12.78*	4.58*
C_max _(μM)	Budesonide	24	1.08^†^	95.9^†^
	Fluticasone	23	0.09^†^	37.9^†^

C_max _= maximum concentration; CV = coefficient of variation; MRT = mean residence time; SD = standard deviation; T_max _= time to maximum concentration.

* Arithmetic mean/SD; ^† ^Geometric mean/CV

### Budesonide and fluticasone in expectorated sputum over the 6-hour collection period in COPD patients

**Figure 4:**
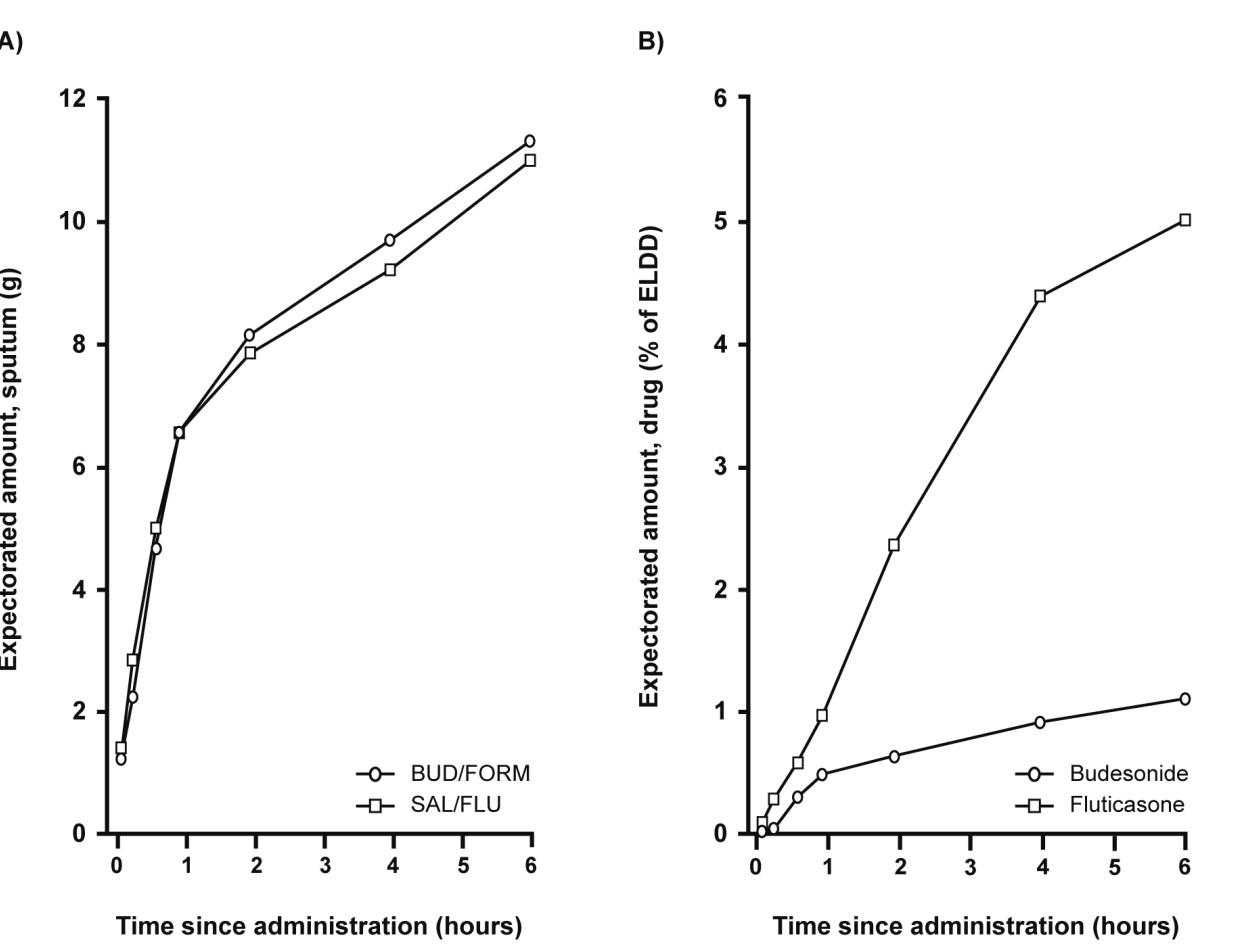
**Cumulative mean amounts of expectorated sputum (A) and budesonide and fluticasone (B) over 6-hour collection**. Mean value plots of the amount of (A) expectorated sputum (arithmetic means) and (B) budesonide and fluticasone in the expectorated sputum (percentage of estimated lung deposited dose [ELDD], geometric mean), cumulative over the 6-hour collection period. UD/FORM = budesonide/formoterol, SAL/FLU = salmeterol/fluticasone.

The average weight of expectorated sputum over the 6-hour collection time period was similar for both treatment periods (Figure 4A). The majority of the expectorated fraction of budesonide was retrieved within the first 2 hours, after which very little was added (Figure 4B). In contrast, fluticasone was continuously expectorated over a longer time period (Figure 4B). The mean expectorated amount of fluticasone (a percentage of ELDD; geometric mean 5.78; 95% CI: 2.59–12.9) was approximately five times higher than budesonide (geometric mean 1.11; 95% CI: 0.52–2.37) over the 6-hour post-dose time period (fluticasone/budesonide: geometric mean 5.21; 95% CI: 1.72–15.8; p = 0.006).

### Relationship between AUC for budesonide and fluticasone, and the amount of drug expectorated and lung function in COPD patients

**Figure 5:**
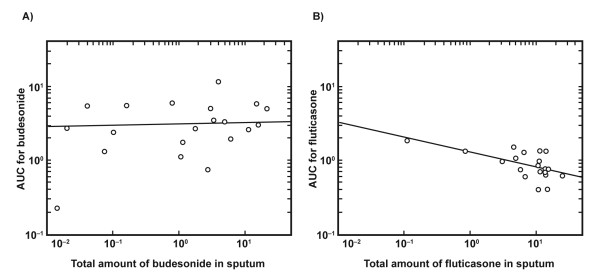
**The relationship between drug exposure and expectorated steroid for budesonide (A) and fluticasone (B)**. Area under the curve (AUC) versus the amount of expectorated ICS. A) Budesonide: p = 0.33; B) fluticasone: p = 0.013 (Spearman's rank correlation test).

**Figure 6:**
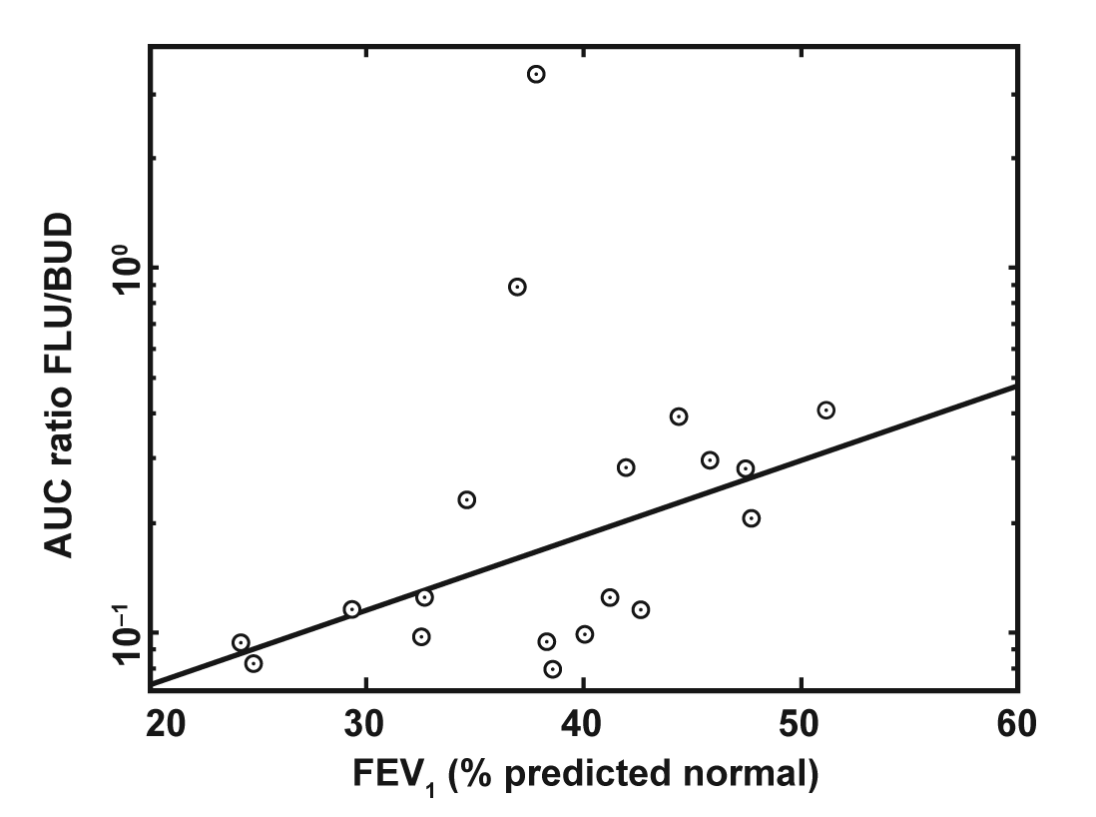
**Dependency of lung obstruction on AUC**. The relationship between area under the curve (AUC) ratio for plasma concentration of fluticasone (FLU) versus budesonide (BUD) and lung function (forced expiratory volume in 1 second [FEV_1_], % predicted normal); p = 0.026 (Spearman's rank correlation test).

There was a tendency for a negative relationship to exist between the amount of expectorated fluticasone and the fluticasone AUC. This was not observed for budesonide (Figure 5). There was also a tendency for the AUC ratio of fluticasone to budesonide to decline at lower FEV_1 _% predicted normal, i.e. AUC for fluticasone decreases relative to budesonide in patients with lower lung function (Figure 6).

## Discussion

This study demonstrated that after inhalation with a LABA, plasma levels of budesonide and fluticasone are lower in patients with severe COPD than in healthy volunteers; however, there is no difference in the AUC ratios between the two steroids. Fluticasone is present in the sputum for longer than budesonide resulting in a higher proportion of the inhaled dose being expectorated in the sputum.

The study did not demonstrate a difference in the ratio of the relative systemic availabilities of inhaled budesonide and fluticasone between healthy subjects and patients with severe COPD. This finding is counter to previous clinical studies that have reported a lower systemic bioavailability of fluticasone, but not budesonide, among patients with marked airway obstruction due to asthma compared with healthy subjects [13,14,18]. These previous observations have been partly attributed to the more central deposition of ICS in obstructed airways and the higher lipophilicity of fluticasone relative to budesonide [10,11]. Both drugs are likely to be deposited more proximally in the obstructive airway but being more lipophilic, fluticasone is less soluble in the airway mucus than budesonide and will therefore be present in the proximal airways for longer and thus, is more likely to be cleared from the airways than budesonide.

Possible reasons for the conflicting results between our study and these previous studies could include the fact that we selected patients with severe COPD (mean 37.5% FEV_1 _predicted normal) and daily sputum production, whereas the aforementioned studies were in subjects with asthma [13,14,18]. This may be of importance given the fact that mucociliary clearance is impaired in COPD due to long-term tobacco smoking [19] and the presence of a compensatory cough mechanism. It can be speculated that uptransport of the lung deposited dose via cough is more rapid than via the slow mucociliary mechanism and that the more rapid cough uptransport in COPD would alleviate the differences between budesonide and fluticasone in the degree of mucociliary clearance compared to asthma. The extent to which long-term smoking affects absorption of inhaled steroids over airway epithelium is not known. A further difference between our study and previous studies is that we combined budesonide and fluticasone with a LABA (formoterol and salmeterol, respectively), whereas previous studies have used ICSs alone [13,14,18,20]. Studies have shown that LABAs can affect mucociliary beat frequency [21-23], potentiate the inhibitory effect of ICSs on mucin secretion [24] and increase mucus hydration [25], although we think these effects are not likely to be seen after a single dose of LABA.

Our data confirmed previously reported differences in the pharmacokinetics of both steroids in the severe COPD population [14,26]. Budesonide was more rapidly absorbed in the airway tissue compared with the highly lipophilic fluticasone as evidenced by a budesonide T_max _of 15.5 minutes compared with 50.8 minutes for fluticasone, which is consistent with its contribution to a more rapid onset of action, as demonstrated when combined to formoterol, by Cazzola and colleagues [27].

The differences in lung disposition could also have been influenced by differences in inhaler device and particle size [28-31]. As reviewed by Newman and Chan [28], particle size and mode of inhalation are two important determinants of the proportion of ICS that is deposited in the respiratory tract. A particle with an aerodynamic diameter of < 5 μm is more likely to be deposited in the bronchi and bronchioles compared with a particle > 5 μm, which is deposited to a higher degree in the mouth and throat [32]. *In vitro *studies have reported the amount of fine particles (aerodynamic diameter < 5 μm) to be more than double with Turbuhaler compared with Diskus [33]. This may correspond to a higher and more peripheral lung deposition of budesonide (via Turbuhaler) compared with fluticasone (via Diskus) [8,29].

A novel observation was the significant difference in the amount of the two ICSs in expectorated sputum. The amount of fluticasone expectorated (percentage of ELDD) was five times higher than for budesonide, supporting our hypothesis that its greater lipophilicity leads to greater airway clearance through mucociliary clearance and/or cough. On average, approximately 6% of ELDD (geometric mean) of inhaled fluticasone was expectorated over the 6 hours after drug administration, whereas most of the 1% of budesonide expectorated was within the first two hours. Whether this finding could result in decreased host defenses and therefore provide an explanation for the increased risk of developing pneumonia, as reported in a number of recent studies with fluticasone alone or in combination with salmeterol, is an intriguing hypothesis and one worthy of further evaluation [6,34-36].

There was a weak inverse relationship between systemic availability, measured as AUC, for fluticasone and the amount expectorated in the sputum; a higher sputum clearance of fluticasone resulted in a lower airway tissue availability. Such a relationship was not observed for budesonide. Spirometry was not conducted directly before each treatment period so as not to affect spontaneous sputum sampling. However, the data suggest that there was a tendency for lower fluticasone AUC relative to budesonide in patients with lower FEV_1 _(% predicted normal), indicating that higher airway obstruction results in lower systemic and lung availability of fluticasone relative to budesonide.

Certain limitations of this analysis should be acknowledged. These include the relatively small sample size and lack of ICS monocomponent treatment to investigate how ICS is handled with and without LABAs, which can increase mucociliary clearance [21-23,37,38]. However, now that combined therapy is recommended for patients with severe COPD, we believe the current study is more clinically relevant. It is also important to note that sputum was collected upon spontaneous expectoration and therefore probably only represents a fraction of the total amount of sputum produced during this period. Thus, the absolute amount of ICS measured in the sputum was likely to be an underestimate, with the remaining sputum being swallowed before expectoration. Nevertheless, differences in expectorated amounts were controlled for through the cross-over study design, and data were reproducible.

## Conclusion

The present study confirmed that plasma levels of both fluticasone and budesonide are lower in subjects with severe COPD but did not demonstrate a difference in the systemic exposure between budesonide and fluticasone in severe COPD patients relative to healthy subjects. In patients with COPD, a larger fraction of fluticasone was recovered in the expectorated sputum than for budesonide, indicating that fluticasone is more extensively cleared from the airways, while budesonide is more rapidly absorbed into the airway tissue.

## Competing interests

The study described in this manuscript was supported by AstraZeneca. TWH has received funding for advisory boards and honoraria for speaker meetings from AstraZeneca, GlaxoSmithKline and Boehringer Ingelheim. TP, TL, LB and SE hold shares in AstraZeneca. TP, LB and SE are full-time employees in the company, and at the time of conduct of the study, TL was also a full-time employee in the company. CD has no competing interests to declare.

## Authors' contributions

CD and TWH contributed to the design and implementation of the study, interpretation of the results and writing of the manuscript. LB, TL, TP and SE gave input to the design of the study, interpretation of the results and discussion, and the manuscript writing.

All authors had complete access to the study report, made final decisions on all aspects of the article and hence are in agreement with, and approve, the final version of the submitted article.
